# Improving traumatic fracture detection on radiographs with artificial intelligence support: a multi-reader study

**DOI:** 10.1093/bjro/tzae011

**Published:** 2024-04-25

**Authors:** Rikke Bachmann, Gozde Gunes, Stine Hangaard, Andreas Nexmann, Pavel Lisouski, Mikael Boesen, Michael Lundemann, Scott G Baginski

**Affiliations:** Radiobotics ApS, Copenhagen, Denmark; Private Ortaca Yucelen Hospital, Turkey; Department of Radiology, Herlev and Gentofte, Copenhagen University Hospital, Denmark; Radiobotics ApS, Copenhagen, Denmark; Radiobotics ApS, Copenhagen, Denmark; Department of Radiology and Radiological AI Testcenter (RAIT) Denmark, Bispebjerg and Frederiksberg, Copenhagen University Hospital, Denmark; Department of Clinical Medicine, Faculty of Health, and Medical Sciences, University of Copenhagen, Denmark; Radiobotics ApS, Copenhagen, Denmark; Virtual Radiologic, Eden Prairie, MN, United States

**Keywords:** fracture detection, artificial intelligence, diagnostic performance, multi-reader study

## Abstract

**Objectives:**

The aim of this study was to evaluate the diagnostic performance of nonspecialist readers with and without the use of an artificial intelligence (AI) support tool to detect traumatic fractures on radiographs of the appendicular skeleton.

**Methods:**

The design was a retrospective, fully crossed multi-reader, multi-case study on a balanced dataset of patients (≥2 years of age) with an AI tool as a diagnostic intervention. Fifteen readers assessed 340 radiographic exams, with and without the AI tool in 2 different sessions and the time spent was automatically recorded. Reference standard was established by 3 consultant radiologists. Sensitivity, specificity, and false positives per patient were calculated.

**Results:**

Patient-wise sensitivity increased from 72% to 80% (*P* < .05) and patient-wise specificity increased from 81% to 85% (*P* < .05) in exams aided by the AI tool compared to the unaided exams. The increase in sensitivity resulted in a relative reduction of missed fractures of 29%. The average rate of false positives per patient decreased from 0.16 to 0.14, corresponding to a relative reduction of 21%. There was no significant difference in average reading time spent per exam. The largest gain in fracture detection performance, with AI support, across all readers, was on nonobvious fractures with a significant increase in sensitivity of 11 percentage points (pp) (60%-71%).

**Conclusions:**

The diagnostic performance for detection of traumatic fractures on radiographs of the appendicular skeleton improved among nonspecialist readers tested AI fracture detection support tool showed an overall reader improvement in sensitivity and specificity when supported by an AI tool. Improvement was seen in both sensitivity and specificity without negatively affecting the interpretation time.

**Advances in knowledge:**

The division and analysis of obvious and nonobvious fractures are novel in AI reader comparison studies like this.

## Introduction

Traumatic extremity fractures are one of the most common causes of emergency visits worldwide.^1–3^ Conventional radiography is the initial diagnostic method for detecting traumatic extremity fractures, as it is easily accessible and available in most emergency rooms and clinics worldwide. A non-negligible percentage of traumatic extremity fractures are undetected on the initial radiographs, mostly due to reporting errors.^4–6^ Under-reading rates are even higher among certain fracture types, such as paediatric cases.^7^^,^^8^ Missed and insufficiently treated fractures can lead to lifelong problems affecting both physical and mental health as well as the socioeconomic status of the patient.^9^

The value of “double-reading” where 2 readers (either 2 radiologists, an emergency department clinician, and a radiologist/reporting radiographer or one radiologist and a reporting radiographer/radiology resident) independently read the same studies to reduce errors and increase sensitivity is hardly debatable, when it comes to patient outcome and satisfaction. Systematic “double-reading” varies a lot between institutions, as some radiology departments consider the benefit to be too low compared to the costs.^10^

Lately, artificial intelligence (AI) decision support tools analysing radiographs, mammography, ultrasound, CT, and MRI have been introduced as a second reader or decision support tool for radiologists, reporting radiographers and radiology trainees to detect various pathologies like fractures, stroke, breast microcalcification, and tumours.^11–14^

Two recent meta-analyses showed that AI tools were noninferior to clinicians for fracture detection on radiographs,^11^^,^^15^ and other studies have shown a significant improvement in diagnostic performance when these tools are used as a decision support.^16–20^

Our aim in this study was to investigate the diagnostic fracture detection performance of readers from various medical groups, including radiographers, nurses, and medical doctors,^21^ all with close affiliation to A&E departments without and with support from a new commercially available AI decision support tool for traumatic fracture detection in the appendicular skeleton on radiographs in patients ≥2 years of age in a clinically acquired dataset.

## Methods

### Ethical considerations

The study was approved by our Institutional Review Board (WCG Clinical, IRB Tracking Number: 20240639), and the need for informed consent was waived due to the retrospective nature of the study. All reader participants signed an informed consent sheet, where they were informed of their rights to withdraw from the study, at all times.

### Study design

The design was a retrospective, fully crossed multi-reader, multi-case study on a balanced dataset acquired in routine practice, with a fracture detection AI support tool as a diagnostic intervention.^21^

The study was conducted in accordance with the STARD 2015 guidelines for reporting diagnostic accuracy studies.^22^

### Data set

The data for this study originated from a US-based teleradiology provider (Virtual Radiologic, Eden Prairie, MN, United States) acquired following a recent trauma. A total number of 340 radiographic examinations were extracted using stratified random sampling from a large pool of >4800 exams not used for AI model development and subsequently evaluated for eligibility by the first author. The sample size was decided with reference to a previous study that investigated a similar AI tool for fracture detection.^18^ The data were stratified according to anatomical region, age, and fracture status until the sample size was reached. For adult patients (years of age ≥21), 40 exams were included for each of the anatomical regions: shoulder/clavicle, arm/elbow, hand/wrist, knee/leg, foot/ankle, and hip/pelvis. For paediatric patients (2≤years of age < 21), 20 exams were included for each of the anatomical regions: shoulder/clavicle, arm/elbow, hand/wrist, knee/leg, and foot/ankle. Within each stratum, the aim was that 50% of the exams contained at least one fracture, if possible.^23^ Exams were excluded, according to the protocol, if there was the presence of orthopaedic implants, presence of cast or splints, low-quality imaging (eg, insufficient exposure, motion artefact, inappropriate or inadequate number of projections) if exams contained fractures in anatomies outside of the intended use (eg, rib, spine), duplicate images (eg, 2 lateral images) projections within an exam, and exams containing images from multiple anatomical subgroups.

The fracture-positive exams were classified as obvious or nonobvious by the first author (10 years of reporting experience). Obvious fractures referred to clearly displaced, angulated, comminuted, or otherwise readily identifiable fractures, whereas nonobvious fractures referred to nondisplaced and subtle fractures as well as multiple fractures in the same exam to embrace possible satisfaction of search errors as “hard cases”.

### Reference standard

The reference standard was established by 2 consultant musculoskeletal (MSK) radiologists with 1 year (S.H.) and 10 years (G.G.) of postspecialty experience who independently interpreted all exams and marked all fractures with a bounding box, including a unique fracture-ID. Completely healed and chronic fractures were ignored. Each radiologist had access to clinical referral notes and the original radiology reports. Agreement was obtained when both interpreted the exam as “No fracture,” or when all individual bounding boxes had a sufficient overlap, measured as having at least 25% Intersection over Union. Cases of disagreement were adjudicated by a third consultant radiologist (S.G.B., 14 years of postspecialty experience).

### Participants (readers)

Readers were recruited by a campaign on the online professional network LinkedIn. The readers were from different Danish hospitals and included: 2 advanced trauma care nurses (>20 years of experience, of which >10 years in A&E), 3 diagnostic radiographers (8-11 years of clinical experience), 4 A&E trainees (0-3 years of clinical experience in A&E, equivalent to foundation or core training), 3 orthopaedic specialty registrars (4-7 years of clinical experience), and 3 radiology specialty registrars (5-6 years of clinical experience). All readers received written instructions and were onboarded to a browser-based reading platform (V7 Darwin, v7labs, London, United Kingdom), where they completed a training session of 5 cases prior to the study readings. Initially, 3 nurses were included, but 1 withdrew from the study due to technical difficulties.

### Test method

**Figure 1.**
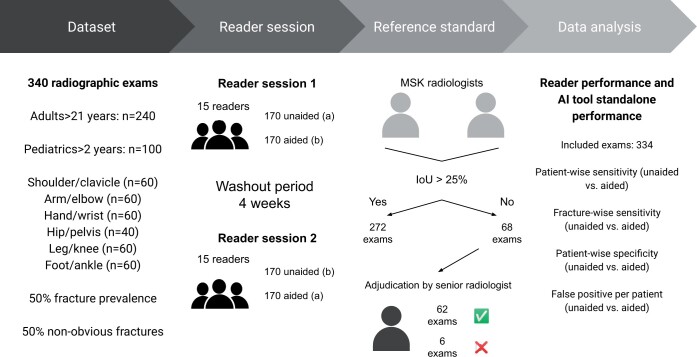
Study workflow.

All 340 exams were evaluated in 2 separate sessions and in 2 different modes: both unaided (control) and aided by the AI tool (experiment), so that each exam was interpreted twice. In the first session, half of the exams contained only the original radiographs (unaided) whereas the other half was supplemented by the output from the AI tool (aided). The second session was separated by at least 4 weeks washout period to reduce memory bias and the reading of unaided/aided exams was swapped^24^ (see Figure 1). The fully crossed design allowed for investigating differences on a patient-level, since all patients were acting as their own control.

As the test was conducted in the reader's spare time, they were allowed to use their own personal computer and there were no requirements for equipment or the physical environment. This reflects how the reading of radiographs in the emergency department is often done, ie, on standard monitors in normal lighting conditions. Each reader interpreted all radiographs in each exam and individual fractures were marked by placing a dot in the centre of a fracture using a point-tool in the reading platform, whereas exams judged as without acute fractures only were marked with a “No fracture” tag, similar to another recent study.^25^ They were blinded to each other's readings, the reference standard, and to their own readings from the first session. Referral notes and original radiology reports were not available to the participants.

Reader session one started April 14, 2023, and the end date for each participant was noted. The average time spent per exam was automatically registered in the reading platform.

### AI tool

The AI tool used in the study (RBfracture™; Radiobotics, Copenhagen, Denmark) is CE-marked as a class IIa medical device and is intended to be used in a clinical setting as a support tool for detecting fractures in the appendicular skeleton on all patients ≥2 years of age. The AI tool is supplied with a Digital Imaging and Communications in Medicine radiograph as input and as output creates a secondary capture and highlights the detected fractures with a box bounding of the findings. The AI tool implements a Deep Convolutional Neural Network based on the Detectron2^26^ framework. The framework is used for generic object detection and was further engineered for the specific task of detecting fractures in radiographic images. The development of the AI tool was done using a dataset consisting of more than 320,000 radiographs collected from more than 100 radiology departments across multiple continents. Prior to development, the data set was split into 3 subsets, a training subset consisting of 80%, a tuning subset of 10%, and an internal verification set consisting of 10% of the data. No image or patient included in the study cohort was used to train or tune the AI tool.

### Statistical analysis

A true-positive (TP) finding was defined as a reader dot located within a reference standard bounding box. A reader dot outside a reference standard bounding box was considered a false positive (FP). A reference standard bounding box without a reader dot was defined as a false negative (FN). An exam marked “No fracture” by a reader and the reference standard was considered a true negative (TN). For the AI tool, a TP was defined when ≥50% of the reference standard bounding box area was covered by the box predicted by the AI tool and conversely, a FN was defined when the coverage was not sufficient (<50%). Boxes from the AI tool not covering at least 50% of a reference standard box were considered FPs. Negative exams not marked by any box by the AI tool were considered TN.

The patient-wise sensitivity was defined as the proportion of patients in whom all fractures were detected (each unique fracture in at least one radiograph) taking multiple fractures within an exam into account. Note that this metric is not influenced by any potential FP predictions. The patient-wise specificity was defined as the proportion of patients in whom no fracture dot was marked among patients without any fracture. The fracture-wise sensitivity was defined as the proportion of TPs amongst all fractures, counting multiple fractures per patient where appropriate. The average number of FP fractures per patient was defined as the total number of FPs divided by the number of patients. CI at the 95% level were estimated by resampling patients with replacement (bootstrap) 1000 times, where the size of the drawn sample was equal to the original sample size. The differences in reader sensitivity, specificity, and average number of FPs were considered significant when the 95% CI did not contain zero.

The changes in reader performance and the standalone performance of the AI tool were evaluated for the different subgroups. In addition, the reader performance was evaluated across reader specialties and for the AI tool, the performance of female and male patients was evaluated.

All the statistical analyses were performed using Python (version 3.8.8, and the libraries Scikit-learn [0.24.1], NumPy [1.23.5], and Scipy [1.6.2]).

## Results

### Data set characteristics

The reference standard was unanimously defined in 272 exams and adjudicated in 68 exams. Six studies were excluded due to disagreements among all 3 consultant radiologists. The excluded studies were shoulder (*n* = 1), ankle (*n* = 2), knee (*n* = 2), and hand (*n* = 1). Of the included patients, 136 were females, 106 were males, and information about patient sex was missing on the remaining 92 patients. Data set characteristics for the included 334 patients are shown in Table 1.

**Table 1.** Overview of exams included in analysis.

	Obvious fractures	Nonobvious/multiple fractures	No fractures	Total
Shoulder and clavicle	20 (13/7)	9 (6/3)	30 (20/10)	59 (39/20)
Elbow and arm	13 (8/5)	16 (12/4)	31 (20/11)	60 (40/20)
Hand and wrist	14 (10/4)	16 (10/6)	29 (19/10)	59 (39/20)
Hip and pelvis	11 (11/0)	9 (9/0)	20 (20/0)	40 (40/0)
Knee and leg	12 (10/2)	14 (9/5)	32 (20/12)	58 (39/19)
Foot and ankle	11 (8/3)	19 (13/6)	28 (18/10)	58 (39/19)
Total	81 (60/21)	83 (59/24)	170 (117/53)	334 (236/98)

Obvious fractures refer to clearly displaced, angulated, comminuted, or otherwise readily identifiable fractures. Numbers in parentheses refer to the number of exams for the adult/paediatric subgroups, respectively.

### Reader performance

**Figure 2.**
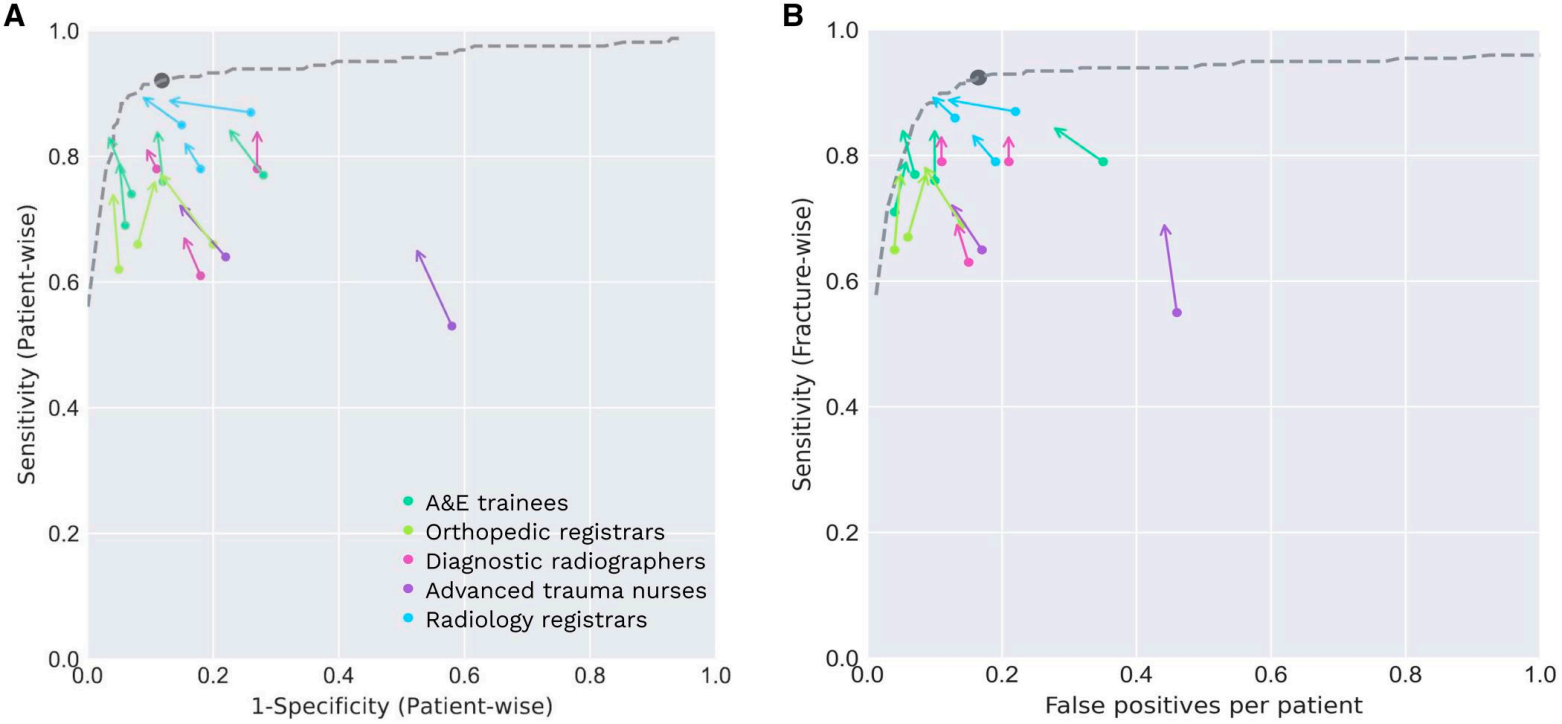
ROC and free-response receiver operating characteristic curves are shown in (A) and (B), respectively. The coloured circles show the unaided performance and the arrow-end show the aided performance for each reader. Arrows pointing towards the upper left corner indicate readers who improved both the sensitivity and specificity. The dashed lines were constructed by artificially varying the probability threshold of the AI tool. The solid grey circles represent the standalone performance of the AI tool. The ROC-AUC was 0.94 (CI, 0.90-0.97). Abbreviations: ROC-AUC = area under the ROC curve; ROC = receiver operating characteristic.

The average patient-wise sensitivity across all readers increased from 72% (CI, 70%-73%) in the unaided readings to 80% (CI, 78%-82%) when reading with aid from the AI tool. The average patient-wise specificity increased from 81% (CI, 80%-83%) to 85% (CI, 84%-87%). Similar results were observed for the fracture-wise sensitivity increase and decrease in number of FP per patient (Table 2 and Figure 2).

**Table 2.** Average diagnostic performance of 15 readers unaided and aided by AI.

	Unaided	Aided	Pairwise difference
Patient-wise sensitivity	0.72 (0.70-0.73)	0.80 (0.78-0.82)	**0.08 (0.06-0.11)**
Patient-wise specificity	0.81 (0.80-0.83)	0.85 (0.84-0.87)	**0.04 (0.02-0.06)**
Fracture-wise sensitivity	0.73 (0.72-0.75)	0.81 (0.79-0.82)	**0.08 (0.05-0.10)**
False positives per patient	0.16 (0.15-0.17)	0.14 (0.13-0.15)	**−0.02 (−0.04 to −0.01)**

Numbers in parentheses are the 95% CI. Boldface pairwise differences indicate *P *<* *.05.

**Figure 3.**
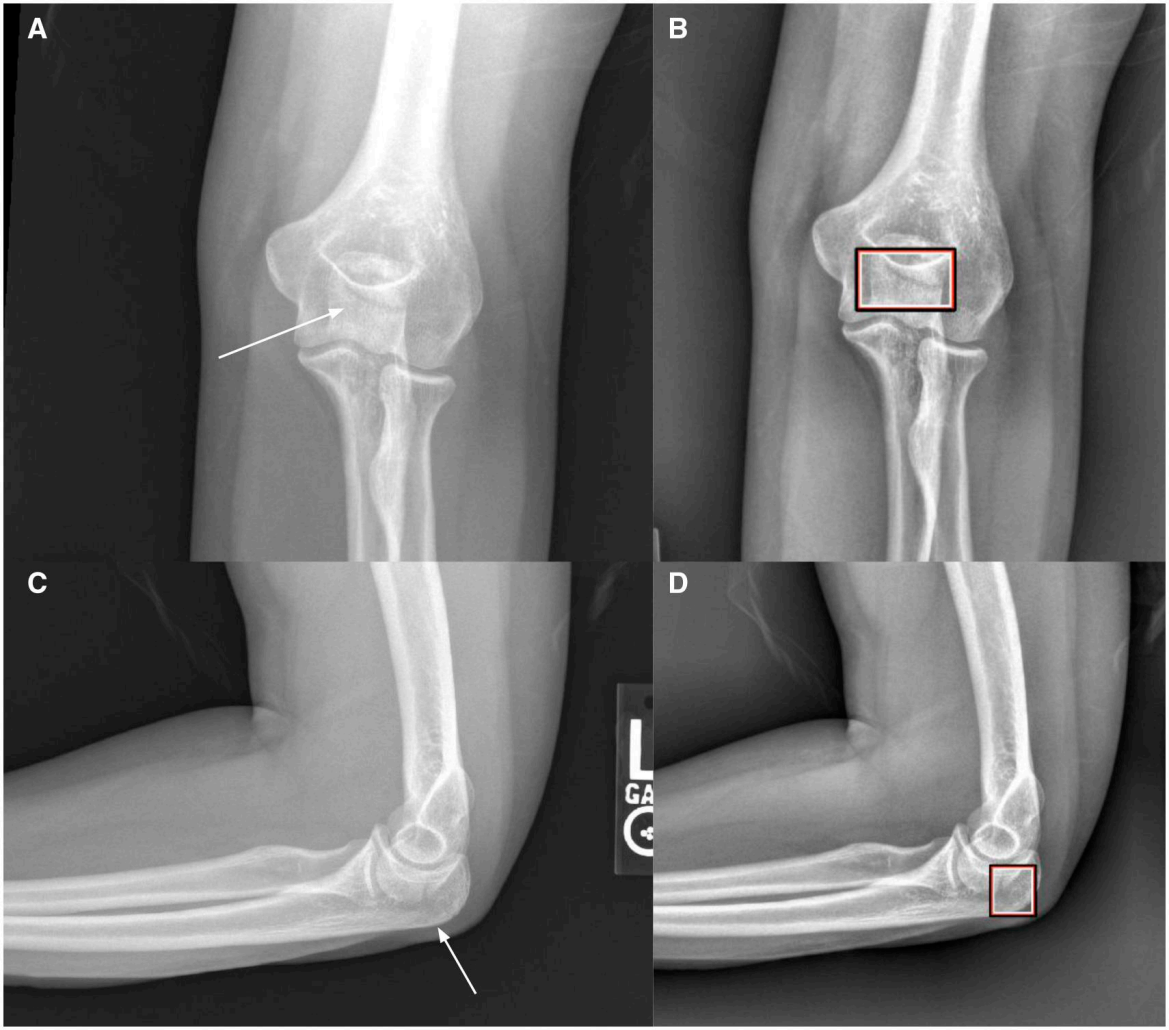
Anterior-posterior (A) and lateral (C) radiographs of nondisplaced fracture of the olecranon (white arrows). The fracture was missed by 11 readers in the unaided exam, but detected by all readers in the aided exam, with support from the AI tool (B, D).

The readers had an average overall relative reduction of missed fractures of 29% in the aided exams (38 missed fractures), compared to the unaided exams (53 missed fractures) (Table 3). An example of an elbow fracture that was missed by 11 readers in the unaided reading, but found by all readers when aided by the AI tool is shown in Figure 3. The average number of FP diagnoses was reduced from 32 to 26, a relative reduction of 21%. The largest improvements in fracture detection were seen for the A&E trainees and orthopaedic registrars with 33% and 35% decreases in missed fractures, respectively. The radiology registrars had the lowest number of missed fractures and also the smallest reduction in number of missed fractures (21%) with support from the AI tool (25 missed fractures) compared to the unaided exams (31 missed fractures) (Table 3). All results from the different reader groups are shown in Table S1.

**Table 3.** Reduction in missed fractures with AI aid.

	Missed fractures unaided	Missed fractures aided	Relative reduction in missed fractures (%)
All readers	53.1 [26-89]	37.8 [20-60]	29
Radiology registrars	31.3 [26-41]	24.7 [20-32]	21
Orthopaedic registrars	65.3 [61-70]	42.7 [41-43]	35
A&E trainees	47.8 [42-57]	32.2 [30-39]	33
Diagnostic radiographers	52.3 [41-74]	41.0 [31-59]	22
Advanced trauma nurses	79.0 [69-89]	56.5 [53-60]	29

Numbers reported are the average [range] across readers.

No significant difference was found in the average reading time for unaided (49 s per exam) and aided (46 s per exam) exams (*P* > .05).

### Subgroup analyses

**Figure 4.**
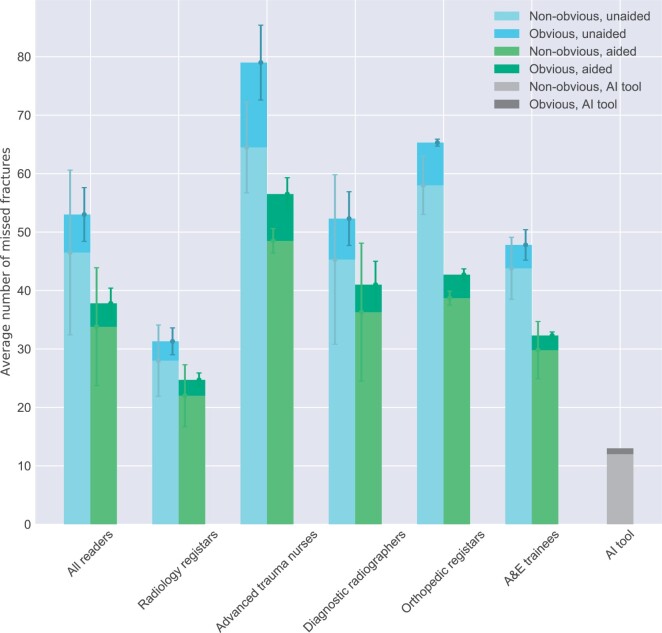
Stacked bar plot showing the average number of missed fractures for each professional group and the AI tool, in unaided and aided nonobvious fractures as well as unaided and unaided obvious fractures. Error bars show the measure of spread (±1 SD) from the average values. Abbreviation: AI = artificial intelligence.

The largest gain from reading in the aided mode was observed on nonobvious fractures where the patient-wise sensitivity increased significantly by 11 pp (CI, 8-14) as compared to the obvious fractures where the increase was only 3 pp (CI, 1-5), but still statistically significant. The average number of missed obvious and nonobvious fractures from both the unaided and aided reading for the different reader groups and the AI tool is shown in Figure 4.

**Figure 5.**
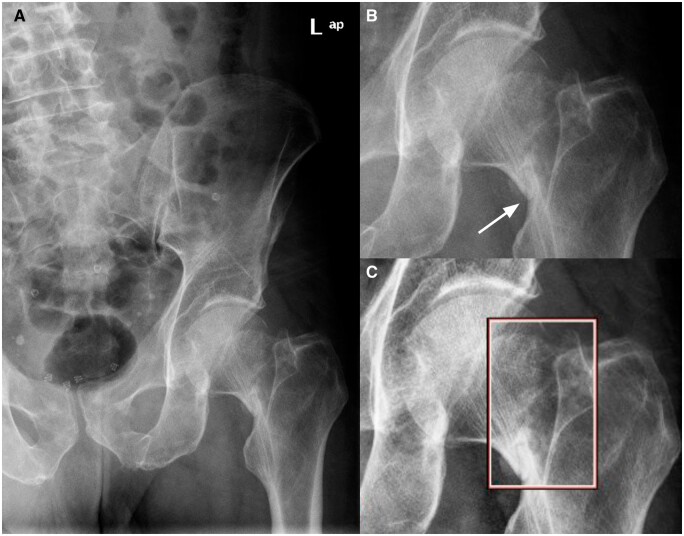
Anterior-posterior radiograph (A) of a nonobvious hip fracture unaided. The subcapital hip fracture (B, white arrow) was missed by 9 readers in the unaided exam, but detected by all readers in the aided exam, with support from the AI tool (C).

The changes in diagnostic performance for the various anatomical regions are shown in Table 4. The largest improvement in fracture detection was seen in hip and pelvis examinations, where the sensitivity significantly increased by 14 pp (CI, 9-20). One example of this is shown in Figure 5. On average, the readers showed the lowest unaided patient-wise sensitivity in ankle and foot exams at 62% (CI, 58%-67%) which increased significantly to 74% (CI, 69%-78%) with AI tool support (Table 4).

**Table 4.** Diagnostic performance for different anatomical regions.

	Unaided	Aided	Pairwise difference
**Shoulder and clavicle (*n* = 59)**			
Patient-wise sensitivity	0.87 (0.84 to 0.90)	0.91 (0.88 to 0.93)	0.04 (0.00 to 0.07)
Patient-wise specificity	0.83 (0.80 to 0.87)	0.84 (0.81 to 0.88)	0.01 (−0.03 to 0.06)
Fracture-wise sensitivity	0.88 (0.84 to 0.90)	0.91 (0.88 to 0.94)	0.04 (0.00 to 0.07)
False-positive per patient	0.13 (0.11 to 0.15)	0.13 (0.11 to 0.16)	0.00 (−0.03 to 0.04)
**Elbow and arm (*n* = 60)**			
Patient-wise sensitivity	0.64 (0.59 to 0.69)	0.69 (0.65 to 0.74)	0.05 (−0.01 to 0.12)
Patient-wise specificity	0.76 (0.72 to 0.80)	0.89 (0.86 to 0.92)	**0.14 (0.09 to 0.18)**
Fracture-wise sensitivity	0.71 (0.68 to 0.75)	0.76 (0.72 to 0.79)	0.04 (−0.01 to 0.09)
False-positive per patient	0.18 (0.15 to 0.20)	0.10 (0.07 to 0.12)	−**0.08 (**−**0.11 to** −**0.04)**
**Hand and wrist (*n* = 59)**			
Patient-wise sensitivity	0.67 (0.63 to 0.72)	0.76 (0.73 to 0.80)	**0.09 (0.03 to 0.15)**
Patient-wise specificity	0.83 (0.80 to 0.87)	0.84 (0.81 to 0.88)	0.01 (−0.04 to 0.07)
Fracture-wise sensitivity	0.73 (0.69 to 0.76)	0.80 (0.77 to 0.83)	**0.08 (0.03 to 0.12)**
False-positive per patient	0.16 (0.13 to 0.19)	0.16 (0.13 to 0.18)	0.00 (−0.04 to 0.04)
**Hip and pelvis (*n* = 40)**			
Patient-wise sensitivity	0.78 (0.73 to 0.82)	0.92 (0.89 to 0.95)	**0.14 (0.09 to 0.20)**
Patient-wise specificity	0.86 (0.81 to 0.90)	0.87 (0.83 to 0.91)	0.01 (−0.05 to 0.06)
Fracture-wise sensitivity	0.78 (0.73 to 0.82)	0.92 (0.89 to 0.95)	**0.14 (0.09 to 0.20)**
False-positive per patient	0.11 (0.09 to 0.14)	0.12 (0.09 to 0.14)	0.00 (−0.04 to 0.04)
**Knee and leg (*n* = 58)**			
Patient-wise sensitivity	0.73 (0.69 to 0.77)	0.82 (0.78 to 0.86)	**0.09 (0.03 to 0.15)**
Patient-wise specificity	0.82 (0.79 to 0.86)	0.85 (0.82 to 0.88)	0.3 (−0.02 to 0.08)
Fracture-wise sensitivity	0.72 (0.67 to 0.76)	0.81 (0.77 to 0.85)	**0.09 (0.03 to 0.15)**
False-positive per patient	0.17 (0.14 to 0.20)	0.13 (0.11 to 0.16)	−0.04 (−0.08 to 0.00)
**Foot and ankle (*n* = 58)**			
Patient-wise sensitivity	0.62 (0.58 to 0.67)	0.74 (0.69 to 0.78)	**0.12 (0.06 to 0.18)**
Patient-wise specificity	0.79 (0.75 to 0.83)	0.81 (0.78 to 0.85)	0.3 (−0.02 to 0.08)
Fracture-wise sensitivity	0.64 (0.60 to 0.68)	0.74 (0.70 to 0.78)	**0.10 (0.05 to 0.16)**
False-positive per patient	0.21 (0.17 to 0.24)	0.18 (0.15 to 0.21)	−0.03 (−0.07; 0.02)

Numbers in parentheses are the 95% CI. Boldface pairwise differences indicate *P *<* *.05.

**Figure 6.**
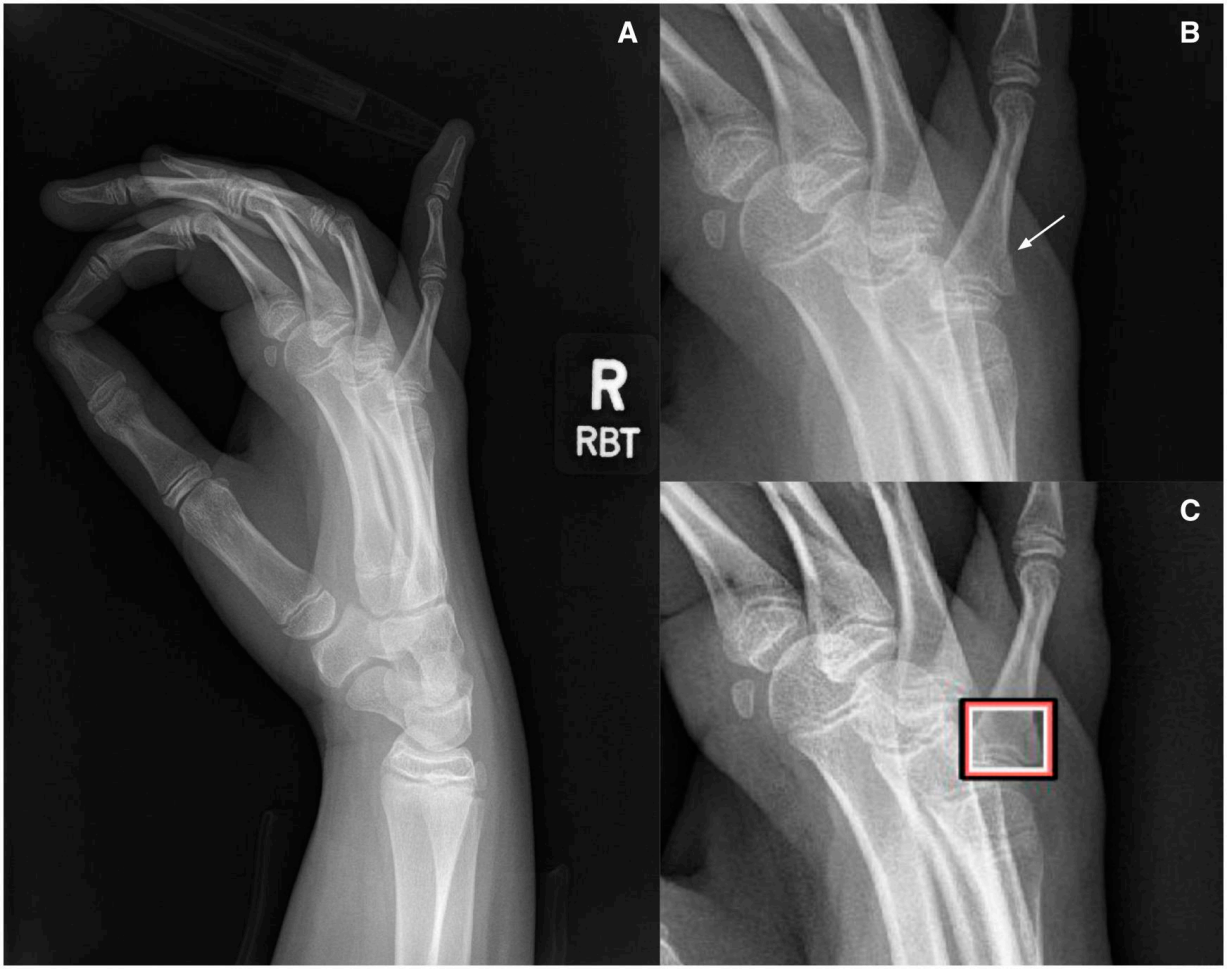
A lateral paediatric hand radiograph (A) with a Salter Harris type II fracture of the base of fifth proximal phalanx (B, white arrow). The fracture was detected by the AI tool (C). The fracture was missed by 11 readers in the unaided exam, but detected by 14 readers in the aided exam with support from the AI tool.

The increase in reader sensitivity was significant regardless of patient age group (adult vs paediatric) (Table S2). The sensitivity for the unaided reading was higher for the paediatric group (78%, CI, 74%-81%) compared to the adult group (69%, CI, 67%-71%), but despite this, the largest gain in patient-wise sensitivity was observed for the paediatric group. An example of a paediatric fracture missed by most of the readers is shown in Figure 6. A significant increase in patient-wise specificity and a decrease in the number of false positives per patient were only seen for the adult subgroup (Table S2).

### Standalone AI performance

**Figure 7.**
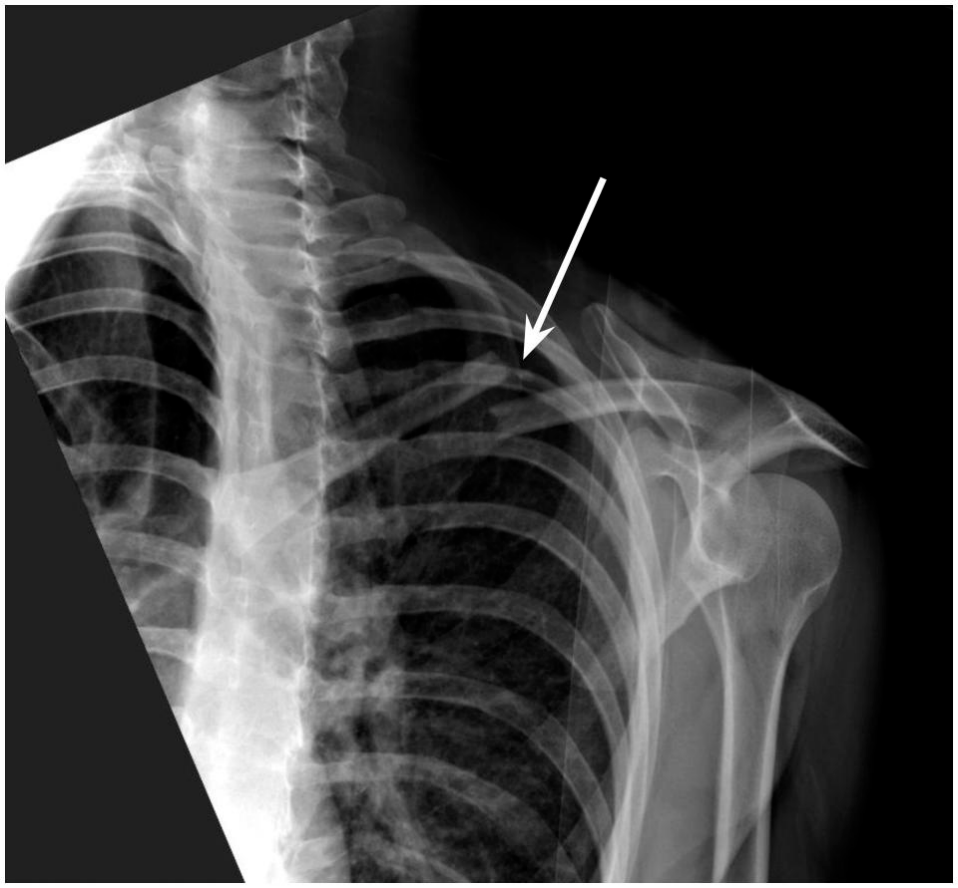
An anterior-posterior radiograph of the left shoulder and clavicle of a 30-year-old woman. A markedly displaced fracture of the clavicle (white arrow) was missed by the AI tool. The fracture was detected by 11 readers in the unaided session and 14 readers in the aided session.

The AI tool showed a standalone patient-wise sensitivity of 92% (CI, 88%-96%) and a patient-wise specificity of 88% (CI, 83%-92%). The fracture-wise sensitivity was 93% (CI, 88%-96%) and the number of false positives per patient 0.16 (CI, 0.12-0.22) (Table 5 and Figure 4). The AI tool excelled in detecting hand/wrist and hip/pelvis fractures with a sensitivity of 100% (CI, 100%-100%) (Table 5). The number of FP per patient was highest for wrist/hand and foot/ankle. The AI tool showed the lowest patient-wise sensitivity for foot/ankle and knee/leg (Table 5). An example of a fracture that was missed by the AI tool is visualized in Figure 7. The fracture was detected by 14 of the 15 readers in the aided session.

**Table 5.** Standalone performance in relevant patient subgroups.

	Patient-wise sensitivity	Patient-wise specificity	Fracture-wise sensitivity	False positives per patient
**Overall performance**				
All patients (*n* = 334)	0.92 (0.88-0.96)	0.88 (0.83-0.92)	0.93 (0.88-0.96)	0.16 (0.12-0.22)
**Patient sex**				
Female (*n* = 136)	0.88 (0.80-0.95)	0.88 (0.80-0.95)	0.89 (0.80-0.96)	0.18 (0.10-0.26)
Male (*n* = 106)	0.89 (0.80-0.98)	0.88 (0.79-0.95)	0.91 (0.83-0.98)	0.16 (0.08-0.25)
**Age**				
Adult (*n* = 236)	0.89 (0.83-0.95)	0.92 (0.87-0.97)	0.90 (0.85-0.95)	0.14 (0.19-0.20)
Paediatric (*n* = 98)	1.00 (1.00-1.00)	0.79 (0.67-0.89)	1.00 (1.00-1.00)	0.22 (0.12-0.34)
**Body part**				
Shoulder/clavicle (*n* = 59)	0.93 (0.83-1.00)	0.93 (0.83-1.00)	0.93 (0.84-1.00)	0.10 (0.02-0.22)
Elbow/arm (*n* = 60)	0.90 (0.78-1:00)	0.93 (0.83-1.00)	0.93 (0.85-1.00)	0.10 (0.03-0.18)
Hand/wrist (*n* = 59)	1.00 (1.00-1.00)	0.80 (0.63-0.93)	1.00 (1.00-1.00)	0.22 (0.10-0.37)
Hip/pelvis (*n* = 40)	1.00 (1.00-1.00)	0.90 (0.75-1.00)	1.00 (1.00-1.00)	0.10 (0.02-0.20)
Knee/leg (*n* = 58)	0.88 (0.75-1.00)	0.87 (0.74-0.97)	0.89 (0.77-1.00)	0.18 (0.05-0.33)
Foot/ankle (*n* = 58)	0.83 (0.69-0.96)	0.86 (0.73-0.97)	0.93 (0.68-0.96)	0.27 (0.12-0.43)
**Fracture type**				
Obvious (*n* = 81)	0.99 (0.96-1.00)	N/A	0.99 (0.96-1.0)	N/A
Nonobvious (*n* = 83)	0.86 (0.78-0.93)	N/A	0.88 (0.82-0.95)	N/A

## Discussion

This study investigated how the use of an AI tool for fracture detection changed the diagnostic performance of A&E nurses, radiographers, and medical doctors. Our results showed a significant reader improvement in fracture detection sensitivity of 8 percentage points, which was equivalent to a 29% reduction in the number of missed fractures in this study. The patient-wise specificity improved by 4 percentage points which in our study meant 21% less overcalls in fracture diagnosis.

Missed fractures on conventional radiographic imaging remain a challenge in acute A&E settings and are one of the most common areas of diagnostic errors in clinical practice.^2^^,^^4–6^^,^^27^ In the 2013 study by Whang et al, among malpractice claims against radiologists, missed nonspinal fractures were found to be the second most common after missed breast cancers.^28^ Diagnostic errors as missed fractures can occur due to different reasons.^9^^,^^27^^,^^29^^,^^30^ In a study by Kim and Mansfield, under-reading (defined as “where the finding was simply missed”) was found to be the most common (42%) cause of error regarding radiological diagnosis.^30^ Diagnostic errors must be decreased to minimize the number of treatment errors, unnecessary pain and discomfort for the patient, patient-recalls, and potential litigations.^9^^,^^28^

Double-reading is an effective way to increase diagnostic performance and current guidelines from the National Institute of Health and Care Excellence recommend that a radiologist, reporting radiographer or other trained reporter should deliver the definitive written report of emergency department radiographic examinations of suspected fractures before the patient is discharged from the emergency department.^31^ Double-reading is resource-heavy^10^ and not always possible in a conventional clinical setting where the frontline medical staff often needs to assess the radiographs immediately, in particular during “off hours”, to decide possible treatment or discharge of the patient.^32^^,^^33^

Our results are in line with previously published studies^16–19^ and underpin the potential for AI tools to support and improve the performance of clinical readers in the detection of fractures.

Interestingly, the improvement in reader sensitivity was most pronounced for nonobvious fractures illustrating the clinical potential of the current AI tool, as these fractures are easily overlooked in normal practice. Another large improvement was seen for hip and pelvic fractures, regardless of visibility, which is of high importance as acute hip fractures require timely diagnosis and treatment, since surgical delay of only 2 days, doubles the first year mortality rate.^34^ The AI tool showed a standalone sensitivity of 100% and a specificity of 90% for fracture detection in hip and pelvic exams and could thus be a valuable support tool in the A&E departments.

The standalone performance of the AI tool with a sensitivity of 92% (CI, 88%-96%) and a patient-wise specificity of 88% (CI, 83%-92%) was comparable to that of other published studies.^18^^,^^35^^,^^36^ Interestingly, in the current study, the fracture detection capability of the AI tool was higher than any of the clinical readers included, highlighting the potential to work as a second reader in a clinical setting. Despite the increased readers performance, in both sensitivity and specificity, none of the groups reached the same performance level as the AI tool. If the readers fully trusted the results of the AI support tool, their performance would have increased even further. The current state of implemented AI products is still low in most hospitals, which can explain the readers’ relatively low trust in the product.^37^^,^^38^

One of the strengths of this study is the inclusion of radiographic examinations of both adult and paediatric patients acquired during normal clinical practice as well as the novelty in including and introducing obvious and nonobvious fractures, which can minimize possible spectrum biases.^39^ Including a wide range of clinical specialties and experiences shows how an AI support tool can affect the reader performance for potential readers. Another strength was the availability of the referral notes and original radiology reports for the consultant radiologists providing the reference standard. This is a significant advancement compared to previous studies.

A limitation of this study was the retrospective case-control design with the use of balanced data with a fracture prevalence of 50%, cleaned from malprojections and suboptimal image quality that does not reflect the true normal population in an A&E department. A balanced data set can introduce spectrum bias and does not allow reliable estimation of the positive and negative predictive value of the AI tool in a real-life clinical setting which should be explored in future clinical studies, but can be defended as the objective of this study was to test reader performance with and without AI aid.^39^ There were no working environment restrictions for the readers during the image interpretation, which potentially could decrease the reader performance, compared to a radiology department with PACS (Picture Archiving and Communication System) monitors and special lighting. The groups of radiologists and orthopaedic surgeons included only readers in training, as these often serve as the frontline personnel in assessing the fracture status of acute patients. Thus the diagnostic impact on specialized clinicians remains unknown and this should also be investigated in future studies. A learning effect from the fully crossed design cannot be ruled out, however, in this case, the changes in performance observed would be conservative estimates of the effect of the AI tool. Finally, clinical information was not available to the readers during the reading sessions and this could potentially influence their diagnostic performance.

## Conclusion

The diagnostic performance for fracture detection of the appendicular skeleton in adult and paediatric populations on conventional radiographs using the new AI decision support tool was significantly improved across all nonspecialist readers with close affiliation to A&E departments, without affecting specificity and the time spent on evaluating the radiographs. The participants detected 29% more fractures on average and had 21% fewer FP fracture diagnoses with the support from the AI tool. These findings support the clinical use of the AI fracture detection tool as a potential second reader for fracture detection on conventional radiographs in the A&E departments.

## Supplementary Material

tzae011_Supplementary_Data
