# Supplementary material for: Improving traumatic fracture detection on radiographs with artificial intelligence support: a multi-reader study
Source: BJR Open. 2024 Apr 25;6(1):tzae011. doi: 10.1093/bjro/tzae011 (PMC11096271; doi:10.1093/bjro/tzae011)
Supplement: tzae011_Supplementary_Data [file tzae011_supplementary_data.docx]

| Table S1. Average diagnostic performance across reader groups | | | | |
| --- | --- | --- | --- | --- |
| Reader and parameter | | Unaided | Aided | Pairwise Difference |
| Radiology registrars (n=3) | | | | |
|  | Patient-wise sensitivity | 0.83 (0.80;0.86) | 0.87 (0.84;0.90) | 0.04 (0.00;0.09) |
|  | Patient-wise specificity | 0.80 (0.77;0.84) | 0.88 (0.85;0.91) | **0.08 (0.03;0.12)** |
|  | Fracture-wise sensitivity | 0.84 (0.81;0.87) | 0.88 (0.85;0.90) | 0.03 (-0.01;0.08) |
|  | False-positive per patient | 0.18 (0.15;0.21) | 0.11 (0.09;0.13) | **-0.07 (-0.10;-0.03)** |
| Orthopedic registrars (n=3) | | | | |
|  | Patient-wise sensitivity | 0.65 (0.60;0.69) | 0.77 (0.73;0.80) | **0.12 (0.06;0.17)** |
|  | Patient-wise specificity | 0.89 (0.86;0.92) | 0.91 (0.89;0.94) | 0.02 (-0.02;0.06) |
|  | Fracture-wise sensitivity | 0.67 (0.63;0.70) | 0.78 (0.75;0.82) | **0.11 (0.07;0.16)** |
|  | False-positive per patient | 0.08 (0.06;0.10) | 0.08 (0.06; 0.09) | 0.00 (-0.03;0.02) |
| A&E trainees (n=4) | | | | |
|  | Patient-wise sensitivity | 0.74 (0.71;0.78) | 0.83 (0.81;0.86) | **0.09 (0.05;0.13)** |
|  | Patient-wise specificity | 0.87 (0.84;0.89) | 0.90 (0.87;0.92) | 0.03 (-0.01;0.06) |
|  | Fracture-wise sensitivity | 0.76 (0.73;0.79) | 0.84 (0.81;0.87) | **0.08 (0.04;0.12)** |
|  | False-positive per patient | 0.14 (0.12;0.16) | 0.12 (0.10;0.14) | -0.02 (-0.05;0.01) |
| Diagnostic radiographers (n=3) | | | | |
|  | Patient-wise sensitivity | 0.72 (0.68;0.76) | 0.79 (0.75:0.82) | **0.06 (0.01;0.11)** |
|  | Patient-wise specificity | 0.81 (0.78;0.85) | 0.83 (0.79;0.86) | 0.02 (-0.03;0.06) |
|  | Fracture-wise sensitivity | 0.74 (0.70;0.77) | 0.79 (0.76;0.83) | **0.06 (0.01;0.10)** |
|  | False-positive per patient | 0.16 (0.14;0.18) | 0.15 (0.13;0.18) | -0.01 (-0.04;0.03) |
| Trauma care nurses (n=2) | | | | |
|  | Patient-wise sensitivity | 0.58 (0.53;0.64) | 0.70 (0.65;0.75) | **0.12 (0.04;0.19)** |
|  | Patient-wise specificity | 0.60 (0.55;0.65) | 0.67 (0.62;0.72) | 0.07 (0.00;0.14) |
|  | Fracture-wise sensitivity | 0.60 (0.55;0.65) | 0.72 (0.67;0.76) | **0.11 (0.05;0.18)** |
|  | False-positive per patient | 0.31 (0.27;0.35) | 0.28 (0.24;0.32) | -0.03 (-0.09;0.03) |
| Numbers in parentheses are the 95% confidence interval. Numbers in brackets are the range. Boldface pairwise differences indicate *p*<0.05 | | | | |

| Table S2. Improvement in fracture detection on adult and pediatric patients | | | | |
| --- | --- | --- | --- | --- |
|  | | Unaided | Aided | Pairwise Difference |
| Adults (n=236) | | | | |
|  | Patient-wise sensitivity | 0.69 (0.67;0.71) | 0.76 (0.74;0.78) | **0.07 (0.04;0.10)** |
|  | Patient-wise specificity | 0.83 (0.82;0.85) | 0.88 (0.86;0.89) | **0.04 (0.02;0.07)** |
|  | Fracture-wise sensitivity | 0.71 (0.69;0.73) | 0.77 (0.76;0.79) | **0.06 (0.04;0.09)** |
|  | False-positive per patient | 0.15 (0.14;0.17) | 0.12 (0.11;0.14) | **-0.03 (-0.05;-0.01)** |
| Pediatrics (n=98) | | | | |
|  | Patient-wise sensitivity | 0.78 (0.74;0.81) | 0.89 (0.87;0.92) | **0.12 (0.08;0.16)** |
|  | Patient-wise specificity | 0.77 (0.74;0.80) | 0.80 (0.77;0.83) | 0.03 (-0.01;0.07) |
|  | Fracture-wise sensitivity | 0.79 (0.76;0.82) | 0.90 (0.88;0.92) | **0.11 (0.07;0.15)** |
|  | False-positive per patient | 0.19 (0.17;0.21) | 0.18 (0.15;0.20) | -0.02 (-0.05;0.02) |
| Numbers in parentheses are the 95% confidence interval. Numbers in brackets are the range. Boldface pairwise differences indicate *p*<0.05 | | | | |
